# Fatigue in Brazilian patients with Parkinson’s disease

**DOI:** 10.1590/1980-5764-DN-2021-0083

**Published:** 2022-05-13

**Authors:** Daniel Venturino Nassif, João Santos Pereira

**Affiliations:** 1 Universidade do Estado do Rio de Janeiro, Hospital Universitário Pedro Ernesto, Departamento de Neurologia, Rio de Janeiro RJ, Brazil.

**Keywords:** Depression, Disorders of Excessive Somnolence, Fatigue, Parkinson Disease, Depressão, Distúrbios do Sono por Sonolência Excessiva, Fadiga, Doença de Parkinson

## Abstract

Fatigue is a non-motor symptom of high prevalence in Parkinson’s disease (PD); however, it is still unknown and neglected by health professionals.

**Objective:**

This study aimed to demonstrate the prevalence of fatigue in patients with PD after excluding confounding factors, as well as its correlation with clinical and demographic data, and to find its negative impact on the quality of life of these patients.

**Methods:**

A cross-sectional study was carried out with 237 randomly selected patients. According to inclusion and exclusion criteria, we selected 53 patients, who were then submitted to the Fatigue Severity Scale. Clinical and demographic data were also analyzed, comparing them between patients with and without fatigue.

**Results:**

We identified fatigue in 21 (39.62%) patients. Patients with and without fatigue had similar mean scores on the UPDRS-III (p=0.36), equivalent daily dose of levodopa (p=0.94), mean disease duration (p=0.43), and mean age (p<0.99). Fatigued patients had worse quality of life scores (PDQ-39) (p=0.00). We did not observe a correlation between fatigue, duration of illness (r=0.11; p=0.43), age (r=0.00; p=0.99), and UPDRS-III (r=0.20; p=0.16).

**Conclusions:**

Fatigue is a highly prevalent and independent symptom of PD. There is no correlation between age, mean duration of disease, motor impairment, and its presence. It has a negative impact on quality of life.

## RESUMO.

A fadiga é um sintoma não motor de elevada prevalência na doença de Parkinson, no entanto ela ainda é desconhecida e negligenciada por profissionais de saúde.

**Objetivo:**

Demonstrar a prevalência de fadiga em pacientes com doença de Parkinson após a exclusão de fatores de confusão, bem como sua correlação com dados clínicos e demográficos, comprovando seu impacto negativo na qualidade de vida desses pacientes.

**Métodos:**

Foi realizado um estudo transversal com 237 pacientes selecionados aleatoriamente. De acordo com critérios de inclusão e exclusão, escolhemos 53 pacientes, que foram então submetidos à Escala de Gravidade de Fadiga. Analisaram-se também dados clínicos e demográficos, comparando-os entre os pacientes com e sem fadiga.

**Resultados:**

Identificamos fadiga em 21 pacientes (39,62%). Pacientes com e sem fadiga apresentaram pontuação média semelhante na Escala Unificada de Avaliação para Doença de Parkinson (UPDRS-III) (p=0,36), dose diária equivalente de levodopa (p=0,94), tempo médio de duração da doença (p=0,43) e idade média (p<0,99). Pacientes fatigados apresentaram piores índices de qualidade de vida (*Parkinson’s Disease Questionnaire* - PDQ-39) (p=0,00). Não observamos correlação entre fadiga, tempo de doença (r=0,11; p=0,43), idade (r=0,00; p=0,99) e UPDRS-III (r=0,20; p=0,16).

**Conclusões:**

A fadiga é um sintoma de alta prevalência e independente na doença de Parkinson. Não há correlação entre idade, tempo médio de duração da doença, comprometimento motor e sua presença. Possui impacto negativo na qualidade de vida.

## INTRODUCTION

Fatigue is one of the most common and disabling non-motor symptoms, which can affect up to half of patients with Parkinson’s disease (PD)^1^. The case definition and diagnostic criteria for identifying PD-related fatigue were published in 2016^2^. Fatigue is a term widely used in clinical practice, and it can be a normal response to exercise or stress, or it can be a sign of some diseases, like PD. In this sense, fatigue can be considered physiological or pathological. In healthy individuals, fatigue is a physiological reaction to intense and prolonged activity, being predictable and transient and can be relieved with rest without compromising daily activities. In patients with pathological fatigue, the characterization is distinct, with fatigue involving feelings of tiredness at rest, a lack of energy that compromises daily activities, or even loss of vigour^3^.

Identifying other non-motor symptoms that act as confounding factors, such as apathy, depression, and excessive daytime sleepiness (EDS), as well as excluding clinical and fatigue-related medications should be the first step in evaluating these patients^4^. This approach was not common in previous studies and should be standardized after the publication of these most recent recommendations^2^ ^,^ ^4^. Knowledge about the pathophysiology of fatigue is scarce, and its diagnosis in clinical practice is made through validated clinical scales^4^. Unfortunately, there is not enough evidence to apply pharmacological and non-pharmacological therapies; therefore, studies on fatigue are of great importance^4^ ^,^ ^5^.

In this study, we aimed to identify fatigue as a primary non-motor symptom after excluding confounding factors, as well as to identify fatigue as an independent non-motor symptom by observing its prevalence in PD patients, its relationship with clinical and demographic characteristics, and the impact of this symptom in the quality of life.

## METHODS

This is an analytical, cross-sectional observational study carried out in the Movement Disorders Sector of Hospital Universitário Pedro Ernesto, Rio de Janeiro, Brazil. The study was approved by the ethics committee of the coordinating center (CAAE number 67871316.9.0000.5259), and all patients signed an informed consent form.

Outpatients of both genders, aged between 50 and 85 years and with a confirmed diagnosis of PD, were randomly selected during a routinely scheduled medical appointment, according to the diagnostic criteria of the Movement Disorders Society^6^, and who could be using any antiparkinsonian drugs. Patients under the age of 50 years (early-onset PD) may present a cognitive and psychiatric profile that are different from those who aged over 50 years, which could somehow make the study less homogeneous^7^. All patients were examined by the same neurologist, who was also responsible for applying all study scales. The exclusion criteria adopted were as follows: dementia, visual or hearing impairment (inability to apply the clinical scales), clinical conditions related to fatigue, such as untreated hypothyroidism, anemia, lung disease, heart disease, nephropathy or liver disease, decompensated diabetes mellitus, previous head injury, autoimmune disease, previous stroke and chronic infectious diseases; modified Hoehn-Yahr Scale (HYS) ≥4^8^, fatigue-related medications such as hypnotics, beta-blockers, benzodiazepines, muscle relaxants, and antihistamines^4^; depressive symptoms, defined by a score >19 on the Beck-II Depression Inventory (BDI-II)^9^; EDS, defined by a score >10 on the Epworth Sleepiness Scale (ESS)^10^; and apathy, defined by a score ≥14 on the Apathy Scale (AE)^11^. The cutoff points used in the scales in question were defined in previous studies, all validated for application in patients with PD and for the Portuguese language. A noteworthy consideration is that BDI-II scores between 14 and 19 are indicative of mild depressive symptoms, but in this study, we prefer a cutoff score of 19 based on previous studies that have assessed the accuracy of this scale in diagnosing major depression in patients with PD. The BDI-II is a screening test and fatigue is related to major depression, not mild depressive symptoms, thus justifying the choice of the cutoff point^9^.

Demographic and clinical characteristics including sex, age, disease duration, medications in use, levodopa equivalent daily dose (LEDD), and HYS were recorded. The LEDD was calculated using a conversion factor previously described in the literature^12^. Scales validated for patients with PD were used to identify apathy (AE), depression (BDI-II), ESS, and dementia (Mini-Mental State Examination [MMSE], as a cognitive screening test, and *DSM-V* criteria, when applicable). Laboratory tests performed included complete blood count, electrolytes, fasting glucose, liver function, urea, creatinine, and thyroid function, and, if abnormalities were detected according to the exclusion criteria, the participant would be excluded.

In the next phase, all selected participants were evaluated in the on phase, or within 1 h of taking the medication. All underwent the Fatigue Severity Scale (FSS), with those with scores higher than 4 (FSS>4) considered fatigued and those with lower scores (FSS≤4) allocated to the group of patients without fatigue. The FSS was recommended for screening and quantifying the severity of fatigue, which is a scale composed of 9 items with the total score representing the average score of each of the 9 items, resulting in a score range between 1 and 7, higher scores indicate a higher level of fatigue^13^. According to the literature, we used an average score greater than 4 points to define clinically significant fatigue^13^ ^,^ ^14^. To assess the degree of motor impairment, all participants underwent the third part of the UPDRS (UPDRS-III)^15^. To measure the impact of fatigue on quality of life, the 39-item Parkinson’s Disease Questionnaire (PDQ-39)^16^ was used.

Frequency, mean, and standard deviation of the variables will be exposed. To verify if the measures of age, disease duration, LEDD, HYS, UPDRS-III, FSS, MMSE, and PDQ-39 were superior, inferior, or equal between the fatigued or not groups, two tests were used. First, Fisher’s F-test was used to identify the equality of variances. Subsequently, Student’s t-test was used for equal variances or different variances. For all these tests, a significance level of 5% was adopted. Among continuous variables, Pearson’s correlation was used. The χ^2^ test was used to compare gender distribution. SPSS version 18 and Microsoft Excel 2010 software were used to compile the analyses and tests.

## RESULTS

From a population of 237 individuals diagnosed with PD, using data from medical records, initially 155 patients were excluded according to the inclusion and exclusion criteria. The remaining 82 patients were invited to participate in this study. After applying the aforementioned scales and carrying out laboratory tests, 29 participants were excluded, so 53 made up the final sample, who were selected and separated into two groups, according to the score obtained on the FSS, as explained in the “Assessment” section. A total of 32 (60.37%) patients constituted the non-fatigued group (FSS≤4) and 21 patients formed the fatigued group (FSS>4), observing a prevalence of fatigue of 39.62% in the evaluated population (n=53). The mean±standard deviation of the FSS was 5.26±0.85 for the fatigued group and 2.31±1.00 for the non-fatigued group (p=0.00). The study population included more men than women (67.92 vs. 32.07%). The mean age of the sample was 65.13±7.94 years, with a mean duration of disease of 7.45±4.20 years. All patients were on antiparkinsonian therapy and the mean LEDD was 714.45±371.72. The mean MMSE score was 27.28±2.02 points.

**Figure 1.**
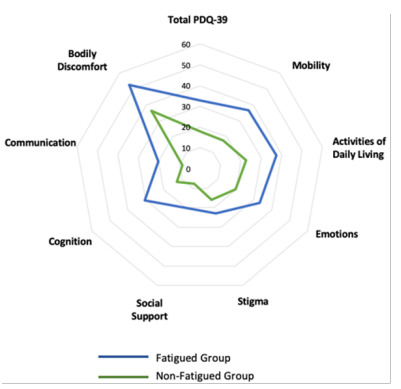
Total Parkinson Disease Questionnaire-39 and domain comparison between fatigued and non-fatigued patients.

**Figure 2.**
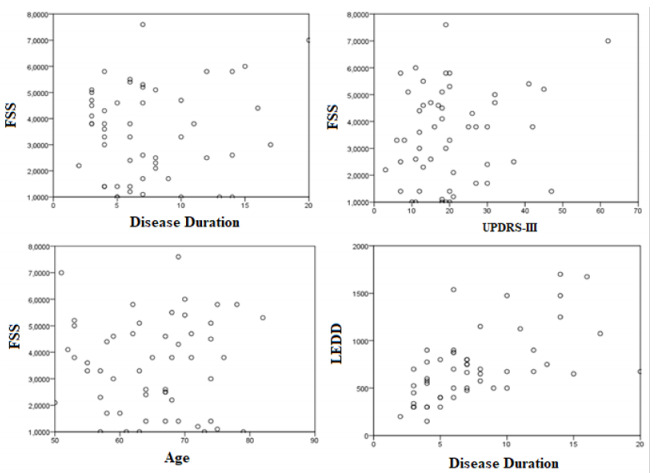
Negative correlation between age, disease duration, Unified Parkinson’s Disease Rating Scale part III, and fatigue.

The fatigued (n=21) and non-fatigued (n=32) groups had the same proportion of male and female patients (χ^2^=0.03; p=0.87). Still comparing the groups, we observed that the patients had a mean age (64.75±7.23 vs. 65.71±8.72; p=0.99), disease duration (7.25±3.64 vs. 7.76±4.82; p=0.43), LEDD (711.06±353.66 vs. 719.61±389.24; p=0.94), and UPDRS-III (19.18±10.34 vs. 22.23±13.14; p=0.36) were similar. Not surprisingly, fatigued patients had worse quality of life scores on the PDQ-39 total score (32.87±12.71 vs. 18.11±13.21; p=0.00) and in all dimensions, except “stigma,” as shown in Table 1 and Figure 1. No correlation was identified between disease duration (r=0.11; p=0.43), age (r=0.00; p=0.99), and UPDRS-III (r=0.20; p=0.16) with the presence of fatigue. Disease duration correlated with LEDD (r=0.59; p=0.00) (Figure 2).

**Table 1.** Clinical and demographic characteristics and Parkinson Disease Questionnaire-39 comparisons between groups.

Clinical and demographic characteristics						
		Non-fatigued (FSS≤4) n=32		Fatigued (FSS>4) n=21		
Sex (male:female), %		68.75:31.25		66.67:33.33		
Age, years, mean±SD		64.75±7.23		65.71±8.72		
HYS (n)	Stage 2	14		8		
	Stage 2.5	11		7		
	Stage 3	7		6		
**Groups comparisons**						
**Variable, mean±SD**		**Non-fatigued (FSS≤4)** **n=32**	**Fatigued (FSS>4)** **n=21**	**95%CI**		**p-value**
				**Lower**	**Upper**	
LEDD		711.06±353.66	719.61±389.24	-203.05	220.17	0.94
MMSE		27.41±1.86	27.10±228	-1.46	0.84	0.59
UPDRS-III		19.18±10.34	22.23±13.14	-3.58	9.68	0.36
Disease duration		7.25±3.64	7.76±4.82	-1.88	2.90	0.43
**PDQ-39**						
**PDQ-39 domain, mean±SD**		**Non-fatigued (FSS≤4)** **n=32**	**Fatigued (FSS>4)** **n=21**	**95%CI**		**p-value**
				**Lower**	**Upper**	
Total		18.1 ±13.21	32.87±12.71	7.29	22.25	0.00*
Mobility		17.73±19.03	36.54±24.05	6.65	30.98	0.00*
Activities of daily living		22.52±20.69	37.30±23.23	2.28	27.27	0.02*
Emotions		19.92±14.57	33.13±20.67	3.30	23.13	0.01*
Stigma		16.01±23.43	22.91±19.22	-5.66	19.47	0.28
Social support		7.81±14.11	20.23±19.51	2.16	22.69	0.02*
Cognition		12.85±15.41	30.65±14.92	9.05	26.55	0.00*
Communication		8.62±12.56	20.23±16.77	2.72	20.51	0.01*
Bodily discomfort		36.19±23.71	52.77±22.76	3.16	30.00	0.02*

SD: standard deviation; 95%CI: 95% confidence interval; HYS: Hoehn-Yahr Scale; UPDRS-III: Unified Parkinson’s Disease Rating Scale part III; PDQ-39: Parkinson Disease Questionnaire-39; LEDD: Levodopa Equivalent Daily Dose; MMSE: Mini-Mental Status Examination; *statistically significant.

## DISCUSSION

Estimated data on the prevalence of fatigue in PD patients vary widely in the literature, around 33-58%^1^ ^,^ ^3^ ^,^ ^4^. This is due to the different criteria adopted for case selection and assessment methods used, as well as the definition of fatigue, which it is still heterogeneous in the literature. This reinforces the need to use similar criteria to define fatigue cases, the most recent being defined by the Movement Disorders Society^2^. We observed a prevalence of fatigue in 39% of our evaluated patients. However, our study sought to eliminate confounding factors, that is, factors other than PD that could be associated with fatigue, showing a more crystalline result and corroborating the concept that fatigue is a primary or independent symptom in PD. It is important to know that the coexistence of non-motor symptoms such as anxiety, EDS, depression, and fatigue is very common, with up to 59% of patients presenting two or more of these symptoms, that is, in addition to overestimating fatigue prevalence data, if these factors of confusion are not removed, the therapeutic approach will be impaired, as the treatment of depressive disorder, for example, is associated with a reduction in fatigue^17^. Another important observation is that these non-motor symptoms associated with fatigue are extremely common in PD. According to literature data, up to 35% of patients with PD have depressive symptoms (17% of them with a diagnosis of major depression)^18^, 17-60%^19^ have apathy, and up to 50% have EDS^20^. So far, especially after the publication of the 2016 case definition recommendations^2^, we did not identify studies with exclusion criteria similar to this one, with the majority having determined the prevalence of fatigue in PD patients without excluding the main causes related to this symptom, and which, as described, are quite common. Thus, we show the high prevalence of fatigue, reinforcing the greater need for health professionals to assess this symptom.

A key point in understanding fatigue as a subjective symptom is to differentiate it from objective fatigue, which is more easily measurable. This point was difficult to understand by the patients evaluated and can also be misinterpreted by health professionals, who commonly confuse fatigue with disability related to the motor symptoms of the disease. An intuitive and mistaken reasoning would be to imagine that the greater the motor impairment, the greater the degree of fatigue; however, fatigue occurs unpredictably in PD and may even be a premotor symptom^21^. It is also important to note that other pathologies that involve the nervous system, such as stroke, even though they do not lead to motor disability, may be associated with fatigue^22^, suggesting alternative mechanisms to the impairment of the motor pathways in the pathophysiology of subjective fatigue. This can be corroborated by neuroimaging studies that show that fatigue in PD is associated with the involvement of non-dopaminergic extrastriatal areas^23^ ^,^ ^24^.

Corroborating the concepts described above, we found similar mean motor impairment scores, through the UPDRS-III, in both groups (FSS>4, 22.23±13.14 vs. FSS≤4, 19.18±10.34; p=0.37) and no correlation between UPDRS-III and fatigue (r=0.20; p=0.16). Likewise, we found similar mean HYS scores between groups (FSS>4, 2.45±0.41 vs. FSS≤4, 2.39±0.38; p<0.59). Similarly, in a recent systematic review, Siciliano et al.^25^ observed, through the UPDRS-III and HYS, a small difference between fatigued and non-fatigued patients, about 5 and 0.33 points, respectively.

We observed a similar mean age between fatigued and non-fatigued patients (FSS>4, 64.75±7.23 vs. FSS≤4, 65.71±8.72, p=0.99), with no correlation between these two variables (r=0.00; p=0.99). As we have sometimes emphasized, fatigue is an independent non-motor symptom that may even precede the motor symptoms of PD, that is, it is part of the pathophysiological process of the disease, which can occur in a wide age range. It is known that with advancing age, chronic conditions associated with fatigue may arise, which we sought to exclude from this study^26^. Studies in the literature that correlated mean age and the presence of fatigue presented varied results. Stocchi et al.^27^ observed a small difference between the mean age (68.0±9.2 vs. 66.3±8.7 years; p=0.044) in fatigued patients compared to non-fatigued patients, the same as observed by Siciliano et al.^25^, through a systematic review by meta-analysis, which observed a mean age 1.7 years higher in fatigued patients (95%CI 0.77-2.12). Alves et al.^28^ also did not observe a statistically significant difference in mean age between the fatigued and non-fatigued groups (74.2±7.9 vs. 72.6±8.8; p=0.216).

In this study, both fatigued and non-fatigued groups had a similar LEDD (FSS>4, 711.06±353.66 vs. FSS≤4, 719.61±389.24; p=0.94). Despite the great benefit that dopaminergic drugs provide in the motor symptoms of PD, other non-dopaminergic mechanisms are probably related to fatigue, such as the decrease in serotonin in the basal ganglia and limbic system^24^. Kang et al.^29^ correlated diffusion tensor imaging values and FSS score in patients with PD and demonstrated that the gray matter volume and striatal dopaminergic activity in PD with fatigue were not different from PD without fatigue, corroborating the involvement of alternative circuits in the pathophysiological process of fatigue. The subclassification of non-motor phenotypes in PD is a relatively recent concept that may result from variable rates of Lewy body deposition and neurodegeneration in different areas of the central nervous system, for example, a higher degree of disease in the limbic system could lead to serotonergic deficit, which is related to a characterized clinical phenotype by fatigue^30^.

Few studies evaluated the impact of dopaminergic drugs in fatigue. The ELLDOPA trial^31^ showed less progression of fatigue in the patient group treated with levodopa than placebo, but it remains unclear if this was a direct effect of levodopa or possibly secondary to other differences such as activity levels. Specialists agree that fatigue does not respond to levodopa in clinical practice^32^. In the RECOVER study^33^, rotigotine, a dopamine agonist with high affinity for all dopaminergic, α-adrenergic, and serotonergic receptor subtypes, improved some non-motor symptoms, such as fatigue, depression, anhedonia, and apathy in patients with PD. Conversely, fatigue can be associated with pramipexole use^34^. In a pilot study with a small sample, rasagiline reduced the levels of fatigue; however, there is a need for further studies to use it for this purpose^35^ ^,^ ^36^. No patients in our study were using this medication. The exact distinction between fatigue and disability generated by motor symptoms is important, as the use of dopaminergic medications will not lead to an improvement in the former and may lead to the appearance of side effects.

Patients in both groups had a similar mean disease duration (FSS<4, 7.25±3.64 vs. FSS≥4.7.76±4.82; p=0.43), with no correlation between this variable and the presence of fatigue (r=0.11; p=0.43).

As already mentioned, fatigue can be a premotor symptom in PD^21^ and some studies have shown its prevalence in newly diagnosed patients with the disease, untreated, and its possibility of progression or appearance over the years. Ongre et al.^37^ observed that these patients had more fatigue than the control subjects, both at baseline and at follow-up after 1 year, showing the precocity of the symptom within the course of the disease. In a 9-year follow-up of the same study, the authors observed an increase or decrease in fatigue levels, as well as the emergence of new cases, showing an unpredictable behavior of this symptom^38^. In the ELLDOPA study^31^, with the same profile of patients, fatigue was identified in one-third of these. In week 42, fatigue still persisted in 50% of patients. Recently, Sciliano et al.^39^ evaluated predictors of fatigue severity in newly diagnosed patients with PD and treatment-naïve over a year and, in addition to observing an initial prevalence of 22%, identified that fatigue can persist and increase over time, with its severity being related to baseline levels of fatigue, apathy, and EDS.

As noted, the presence of fatigue does not depend on the duration of the disease, being observed even in newly diagnosed patients, and it may or may not appear or worsen over the clinical course of the disease. In our study, in accordance with a recent meta-analysis^25^, it was not observed that longer disease duration indicates a higher prevalence of fatigue.

Although the motor symptoms of PD are clearly associated with a negative impact on quality of life, the presence of non-motor symptoms enhances this impact, bearing in mind that, in general, patients with PD have more than one of these symptoms^17^. This study showed that fatigued patients have higher total scores on the PDQ-39, as well as in all domains evaluated, with the exception of the “stigma” domain. These findings are similar to those found by Herlofson et al.^40^, who observed that PD patients with fatigue reported more distress in the dimensions of emotional well-being, mobility, and PDQ summary index and is also in agreement with the study by Okuma et al.^41^, whose results also showed that PDQ total score and score for mobility were significantly associated with fatigue. Other studies, through PDQ-39 and other scales, and a systematic review also corroborated with the negative impact of fatigue in quality of life^25^ ^,^ ^42^.

Among the limitations of this study, we point out the small sample size; however, previous studies that had larger samples did not exclude confounding factors such as this one. Despite this, the result obtained is in accordance with current literature. Other non-motor symptoms that were not evaluated can also interfere with quality of life, so although fatigue is certainly an important negative factor, it is not the only aggravating factor.

Fatigue is still a symptom neglected by health professionals. Its subjectivity, added to the absence of well-established diagnostic criteria and the lack of studies, make its diagnosis and management quite challenging. Future studies should be more homogeneous, as we now have a case definition established in the literature. The search for secondary factors is of fundamental importance, as some are potentially treatable. The distinction between fatigue and motor impairment must be made precisely so that there is no confusion between complaints and inadequate treatment. We saw that its absence in a first evaluation does not exclude the possibility of its appearance in a second moment, as well as several other non-motor symptoms, its behavior being unpredictable. Unfortunately, we still have less information about its pathophysiology and treatment, and this study seeks help in this regard.
